# Effects of Weight Gain after 20 Years of Age and Incidence of Hyper-Low-Density Lipoprotein Cholesterolemia: The Iki Epidemiological Study of Atherosclerosis and Chronic Kidney Disease (ISSA-CKD)

**DOI:** 10.3390/jcm10143098

**Published:** 2021-07-14

**Authors:** Shota Okutsu, Yoshifumi Kato, Shunsuke Funakoshi, Toshiki Maeda, Chikara Yoshimura, Miki Kawazoe, Atsushi Satoh, Soichiro Yokota, Kazuhiro Tada, Koji Takahashi, Kenji Ito, Tetsuhiko Yasuno, Hideyuki Fujii, Shigeaki Mukoubara, Hitoshi Nakashima, Daiji Kawanami, Kosuke Masutani, Hisatomi Arima, Shigeki Nabeshima

**Affiliations:** 1 General Medicine, Fukuoka University Hospital, Fukuoka 814-0180, Japan; shota.o.19870528@gmail.com (S.O.); ykato@fukuoka-u.ac.jp (Y.K.); snabeshi@fukuoka-u.ac.jp (S.N.); 2 Department of Preventive Medicine and Public Health, Fukuoka University, Fukuoka 814-0180, Japan; shunsuke.funakoshi@gmail.com (S.F.); tmaeda@fukuoka-u.ac.jp (T.M.); ychikara@fukuoka-u.ac.jp (C.Y.); miki1024@fukuoka-u.ac.jp (M.K.); atsushis@fukuoka-u.ac.jp (A.S.); 3 Division of Nephrology and Rheumatology, Department of Internal Medicine, Fukuoka University, Fukuoka 814-0180, Japan; syokota@fukuoka-u.ac.jp (S.Y.); ktada@fukuoka-u.ac.jp (K.T.); reply_09030728174@yahoo.co.jp (K.T.); kito@fukuoka-u.ac.jp (K.I.); yasuno9584@fukuoka-u.ac.jp (T.Y.); hitscenenakashima@gmail.com (H.N.); kmasutani@fukuoka-u.ac.jp (K.M.); 4 Department of Endocrinology and Diabetes Mellitus, Fukuoka University School of Medicine, Fukuoka 814-0180, Japan; joeten1cho@yahoo.co.jp (H.F.); kawanami@fukuoka-u.ac.jp (D.K.); 5 Nagasaki Prefecture Iki Hospital, Nagasaki 811-5132, Japan; s-mukoubara@ikihp.jp

**Keywords:** weight gain, LDL cholesterol, hyper-LDL cholesterolemia, longitudinal study, general population

## Abstract

The aim of this study was to investigate the effects of long-term weight gain from the age of 20 on incidence of hyper-low-density-lipoprotein (LDL) cholesterolemia in the general population of Japanese people. Methods: We conducted a population-based retrospective cohort study using annual health checkup data for residents of Iki City, Nagasaki Prefecture, Japan. A total of 3179 adult (≥30 years old) men and women without hyper-LDL cholesterolemia at baseline, who underwent two or more health checkups were included in the analysis. Information on weight gain (≥10 kg) after 20 years of age was obtained using questionnaire. The outcome of this study was development of hyper-LDL cholesterolemia defined as LDL-cholesterol level ≥3.62 mmol/L and/or initiation of lipid-lowering medications. Results: During a mean follow-up period of 4.53 years, 665 of the 3179 participants developed hyper-LDL cholesterolemia (46.5/1000 person-years). The incidence of hyper-LDL cholesterolemia was higher in participants with a weight gain of ≥10 kg (55.3/1000 person-years) than among those with a weight gain of <10 kg (41.8/1000 person-years). This association remained statistically significant even after adjustment for age, sex, smoking, daily drinking, exercise, obesity, hypertension, and diabetes (multivariable hazard ratio 1.31, 95% confidence interval 1.08–1.58, *p* = 0.006). Conclusion: A weight gain of ≥10 after 20 years of age affected the development of hyper-LDL cholesterol regardless of age, sex, and obesity in a general population of Japanese.

## 1. Introduction

Cardiovascular disease is one of the leading causes of death in Japan as well as worldwide, with 18 million fatalities accounting for 32% of total deaths worldwide [1]. The risks of cardiovascular disease are associated with lifestyle factors such as smoking, diet, and exercise habits, and metabolic factors such as obesity, serum lipid levels, hypertension, and diabetes [2,3]. Of these, elevated low-density lipoprotein (LDL) cholesterol levels are among the most important [4]. Randomized controlled trials have demonstrated the short-term effects of interventions to reduce body weight on LDL cholesterol levels [5]. However, the effects of long-term weight changes on LDL cholesterol levels are not clear. The objective of this study was to investigate the effects of long-term weight gain from the age of 20 on incidence of hyper-low-density-lipoprotein (LDL) cholesterolemia in the general population of Japanese.

## 2. Materials and Methods

### 2.1. Study Design and Participants

The Iki Epidemiological Study of Atherosclerosis and Chronic Kidney Disease (ISSA-CKD) project was a retrospective open cohort study of the general population of Iki City, Nagasaki Prefecture, Japan. The details of the study methods have been previously reported [6,7,8,9,10,11]. In brief, Iki Island is in the north of Nagasaki Prefecture and the population is approximately 27,000. In Iki City, medical examinations are conducted annually for residents over 30 years of age. A total of 7895 people who participated in these medical examinations at least once during study period between 2008 and 2017. We excluded 1879 individuals who underwent a health checkup only once, 2289 individuals who had previously been diagnosed with hyper-LDL cholesterolemia (LDL-C levels ≥3.62 mmol/L and/or use of lipid-lowering medications), and 548 individuals with missing information on weight gain after 20 years of age. Therefore, this study enrolled 3179 individuals. The study was approved by the Fukuoka University Clinical Research Ethics Center (approval number: 2017M010).

### 2.2. Data Collection

Information on weight change after 20 years of age was collected using a standardized yes/no questionnaire (“has your weight increased by 10 kg or more since 20 years of age?”). The cut-off point of 10 kg was defined based on recommendation from the Ministry of Health, Labour and Welfare to detect people who gained weight by 10 kg or more after 20 years of age because they are at high risk of diabetes and hypertension [12]. We also used a standardized questionnaire regarding smoking, daily alcohol intake, and regular exercise habits, as well as the use of blood pressure, lipid-lowering, and glucose-lowering drugs. A current smoker was defined as a participant who smoked ≥100 cigarettes or had smoked continuously for ≥6 months. A current alcohol drinker was defined as a participant who drank daily. A regular exercise habit was defined as exercise performed for ≥30 min at least twice weekly. Height and weight were measured without shoes and body mass index (BMI; kg/m^2^) was calculated. Obesity was defined as a BMI ≥ 25 kg/m^2^ [13]. Blood pressure (BP) was measured by trained staff using a mercury, automatic, or aneroid sphygmomanometer with an appropriately sized cuff, measured with the right upper arm according to standard guidelines after at least 5 min of rest in a sitting position [14]. Hypertension was defined as BP ≥ 140/90 mmHg, or use of BP-lowering medications [15]. Casual blood samples were also collected. LDL cholesterol levels were determined using a direct enzymatic method. High-density lipoprotein (HDL) cholesterol and triglyceride levels were also measured using the enzymatic method. Blood glucose and HbA1c levels were measured enzymatically, and diabetes was defined as a fasting blood glucose level ≥7.0 mmol/L, non-fasting blood glucose ≥11.1 mmol/L, HbA1c (National Glycohemoglobin Standardization Program) ≥6.5%, or use of glucose-lowering drugs [16].

### 2.3. Outcome

During the follow-up period from 2008 to 2017, we defined the first time when each participant received a medical examination as the baseline and followed the patients up to 2017. The outcome of this study was incidence of hyper-LDL cholesterolemia. The onset of hyper-LDL cholesterolemia was defined as an LDL-C level of ≥3.62 mmol/L or the initiation of lipid-lowering drugs during the follow-up period [17], which was confirmed at the end of follow-up.

### 2.4. Statistical Analysis

We applied Wilcoxon test for continuous variables and Chi-square tests for categorical variables to compare baseline characteristics between the two groups: those whose weight had increased by ≥10 kg after 20 years of age and those who did not. The incidence of hyper-LDL cholesterolemia was calculated in person-years. The effect of weight gain of ≥10 kg after 20 years of age on the development of hyper-LDL cholesterolemia was estimated using univariable and multivariable Cox proportional hazard models. The multivariable analysis was adjusted for age, sex, smoking, drinking, exercise, obesity, hypertension, and diabetes. We conducted a subgroup analysis to stratify the effect of weight gain on the development of hyper-LDL cholesterolemia by subgroup (under 65 years or over 65 years of age, male or female sex, non-obese or obese). The differences between subgroups were tested by adding an interaction term to the statistical model. Statistical Analysis System (SAS) version 9.4 (SAS Institute Inc., Cary, NC, USA) was used to perform the statistical analyses. All *p*-values reported were two-sided, and the significance level was set at *p* < 0.05.

## 3. Results

Table 1 shows the baseline characteristics according to weight gain after 20 years of age. Participants with a weight gain of ≥10 kg were more likely to be men, obese, hypertensive, and diabetic, as well as higher levels of BMI, blood pressure, HbA1c, HDL-C, LDL-C, and triglycerides. During a mean follow-up period of 4.53 years, 665 of the 3179 participants developed hyper-LDL cholesterolemia (46.5/1000 person-years).

Table 2 shows the risks of hyper-LDL cholesterolemia according to weight gain after 20 years of age. The incidence of hyper-LDL cholesterolemia was higher in participants with a weight gain of ≥10 kg (55.3/1000 person-years) than among those with a weight gain of <10 kg (41.8/1000 person-years). This association remained statistically significant even after adjustment for age, sex, smoking, daily drinking, exercise, obesity, hypertension, and diabetes (multivariable-adjusted hazard ratio 1.31, 95% confidence interval 1.08–1.58, *p* = 0.006). Similar results were obtained after adjustment for more detailed categories for alcohol intake (no drinkers, occasional drinkers or daily drinkers [<22 g/day, 22–43 g/day or ≥44 g/day]): multivariable hazard ratio 1.31, 95% confidence interval 1.08–1.58, *p* = 0.005. Sensitivity analysis with adjustment of BMI instead of obesity demonstrated that the hazard ratio of weight gain ≥10 kg after 20 years of age for development of hyper-LDL cholesterolemia was 1.18 (95% confidence interval 0.97–1.43) but the association was not statistically significant (*p* value = 0.100). Likewise, sensitivity analysis with adjustment of waist circumference instead of obesity demonstrated that the hazard ratio of weight gain ≥10 kg after 20 years of age for development of hyper-LDL cholesterolemia was 1.16 (95% confidence interval 0.96–1.41) but the association was not statistically significant (*p* value = 0.122).

Table 3 shows the results of subgroup analyses. There were no clear differences in the effects of weight gain of ≥10 kg after 20 years of age for the development of hyper-LDL cholesterolemia between subgroups defined by age (<65 vs. ≥65 years), sex, and obesity (all *p* > 0.1 for interaction).

## 4. Discussion

The results of this large-scale longitudinal study of the general Japanese population showed that a weight gain of ≥10 kg after 20 years of age was significantly associated with the incidence of hyper-LDL cholesterolemia. This outcome remained significant even after adjusting for age, sex, smoking, daily drinking, exercise, obesity, hypertension, and diabetes. A similar association was observed between subgroups defined by age, sex, and obesity.

Previous studies have investigated the effects of body weight changes on serum LDL cholesterol levels. A meta-analysis of 73 randomized trials including 32,496 participants (mean age 48 years, weight 102 kg, BMI 36.3 kg/m^2^) reported that short-term body weight reduction due to lifestyle-related interventions was associated with decreased LDL cholesterol levels in both men and women [4]. A prospective observational study of 3388 overweight Polish individuals aged 45–64 years reported was no significant association between a two-year change in body weight and serum LDL cholesterol levels [18]. Regarding the effects of long-term body weight change, a cross-sectional study investigating the association between body weight gain after 20 years of age and the prevalence of hyper-LDL cholesterolemia in 1715 Chinese participants in the general population aged 45–60 years reported that women with a weight gain of ≥10 kg after 20 years of age had a higher prevalence of hyper-LDL cholesterolemia than those without; however, this association was not observed in men [19]. In the present large-scale, longitudinal study of the general Japanese population, long-term body weight change age 20 years of age was associated with increased risks of the future development of hyper-LDL cholesterolemia. The effects of long-term body weight change on hyper-LDL cholesterolemia were comparable between sexes. Based on these findings, long-term weight gain is likely to affect the future development of hyper-LDL cholesterolemia.

The mechanisms underlying the association between long-term weight gain and hyper-LDL cholesterolemia have not been fully elucidated. Weight gain from the age of 20 years appears to mainly reflect an increase in visceral fat volume owing to decreased muscle mass volume and basal metabolic rate after this age [20,21,22]. Increased visceral fat volume causes insulin resistance [23,24,25], which is associated with an increased pool of LDL precursors including very-low-density lipoprotein (VLDL) [26,27]. Insulin resistance is also associated with a reduction in the number of LDL receptors, a decrease in the LDL-binding affinity of these receptors, and a subsequent reduction in the clearance of LDL particles [26,27,28]. Increased pooled LDL precursors and decreased clearance of LDL particles, which are associated with visceral fat, might be attributable to the development of hyper-LDL cholesterolemia.

This is the first longitudinal study of the association between a weight gain of ≥10 kg after 20 years of age and hyper-LDL cholesterolemia in the general population including adult Japanese men and women. The limitations of this study were its retrospective design, the use of a recall-based questionnaire, and the analysis of data from medical examinations of the general public, which may have resulted in a bias toward health-conscious participants.

## 5. Conclusions

In conclusion, in adult Japanese men and women, a weight gain of ≥10 kg after 20 years of age affected the development of hyper-LDL cholesterol regardless of age, sex, and obesity. A population strategy with interventions to maintain a proper weight is important to prevent the subsequent occurrence of cardiovascular events, increased risk of death, and medical costs.

## Figures and Tables

**Table 1:** Participant baseline characteristics according to weight gain after 20 years of age.

	Weight Change after 20 Years of Age		
	<10 kg (*N* = 2146)	≥10 kg (*N* = 1033)	*p* Value
Age	58.9 ± 11.7	58.9 ± 10.4	0.977
Female	1111 (51.8%)	413 (40.0%)	<0.0001
Current smoker	474 (22.1%)	232 (22.4%)	0.810
Daily drinking	574 (26.9%)	331 (32.3%)	0.0018
Exercise	1549 (73.3)	769 (75.2%)	0.250
Body mass index	22.0 ± 2.63	26.1 ± 3.18	<0.0001
Obesity	256 (11.9%)	635 (61.5%)	<0.0001
Systolic blood pressure, mmHg	127 ± 18.9	132 ± 18.4	<0.0001
Diastolic blood pressure, mmHg	73.7 ± 11.0	77.0 ± 11.3	<0.0001
Hypertension	787 (36.7%)	534 (51.7%)	<0.0001
LDL-cholesterol, mmol/L (mg/dL)	2.74 ± 0.54 (106 ± 21.0)	2.90 ± 0.50 (112 ± 19.5)	<0.0001
HDL-cholesterol, mmol/L (mg/dL)	1.67 ± 0.44 (64.6 ± 16.9)	1.46 ± 0.39 (56.5 ± 15.0)	<0.0001
Triglyceride, mmol/L (mg/dL)	1.15 ± 0.87 (102 ± 76.9)	1.56 ± 1.21 (138 ± 107)	<0.0001
HbA1c, %	5.30 ± 0.63	5.50 ± 0.80	<0.0001
Diabetes	133 (6.2%)	106 (10.7%)	<0.0001

LDL, low-density lipoprotein; HDL, high-density lipoprotein; HbA1c_,_ glycated hemoglobin.

**Table 2:** Risks of hyper-LDL cholesterolemia according to weight gain after 20 years of age.

	Weight Change after 20 Years of Age		
	<10 kg (*N* = 2146)	≥10 kg (*N* = 1033)	*p* Value
Number of events	406	259	
Person-years	9721.38	4679.49	
Incidence/1000 person-years	41.8	55.3	
Crude hazard ratio (95%CI)	Reference	1.33 (1.14–1.55)	<0.001
Multivariable-adjusted * hazard ratio (95%CI)	Reference	1.31 (1.08–1.58)	0.006

* Adjusted for age, sex, smoking, daily drinking, exercise, obesity, hypertension, and diabetes. CI, confidence interval.

**Table 3:** Subgroup analyses.

	Adjusted * Hazard Ratio	*p* for Interaction
Age (years)		
<65	1.40 (1.10–1.77)	
>65	1.12 (0.82–1.54)	0.13
Sex		
Male	1.26 (0.96–1.65)	
Female	1.33 (1.02–1.72)	0.90
Obesity		
Absent	1.26 (0.99–1.59)	
Present	1.38 (1.00–1.91)	0.63

* Adjusted for age (except for subgroup analysis by age), sex (except for subgroup analysis by sex), smoking, daily drinking, exercise, obesity (except for subgroup analysis by obesity), hypertension, and diabetes.

## Data Availability

The data are not publicly available in order to preserve the anonymity of the subjects involved in the study.
